# Urban-rural differences in the association between social activities and depressive symptoms among older adults in China: a cross-sectional study

**DOI:** 10.1186/s12877-021-02541-y

**Published:** 2021-10-18

**Authors:** Yanan Wang, Zhen Li, Chang Fu

**Affiliations:** 1 Department of Rehabilitation Treatment, School of Nursing and Health-care, Qingdao Huanghai University, Qingdao, 266555 Shandong China; 2 Qinghai Provincial Center for Diseases Prevention and Control, Xining, 810010 Qinghai China; 3 Department of Health Psychology, School of Nursing and Rehabilitation, Cheeloo College of Medicine, Shandong University, 44 Wenhuaxilu Rd, Jinan, 250012 Shandong China

**Keywords:** Social activity, Depressive symptoms, Older adults, Urban-rural difference

## Abstract

**Background:**

Participation in social activities has positive health effects among older adults; however, few studies have investigated the association between social activity and depressive symptoms among Chinese older adults. This study aimed to examine the association between social activities and depressive symptoms among older adults in China regarding urban-rural differences.

**Methods:**

Data were collected from 8255 respondents from the 2015 China Health and Retirement Longitudinal Study. Depressive symptoms were assessed using the 10-item Center for Epidemiologic Studies Depression Scale. Type and frequency of social activities were collected via a questionnaire. Multivariate logistic regression analysis was used to explore the relationship between social activities and depressive symptoms.

**Results:**

In our study, the prevalence of depressive symptoms was lower in urban older adults compared with rural older adults (25.2% vs. 40.7%). After adjustment for all covariates, our results indicated that interacting with friends almost weekly or almost daily (almost weekly: OR = 0.568, 95%CI: 0.337–0.955; almost daily: OR = 0.664, 95%CI: 0.453–0.973) and participating in community organizations almost daily were inversely associated with depressive symptoms among urban older adults (OR = 0.107, 95%CI: 0.012–0.952). Interacting with friends almost daily (OR = 0.847, 95% CI: 0.720–0.996) and participation in hobby groups either almost every week or almost daily were both inversely associated with depressive symptoms among rural older adults (almost weekly: OR = 0.683, 95%CI: 0.518–0.902; almost daily: OR = 0.567, 95%CI: 0.440–0.731). Participating in sports groups almost daily was inversely associated with depressive symptoms among both urban and rural older adults (urban: OR = 0.664, 95%CI: 0.445–0.991; rural: OR = 0.506, 95%CI: 0.366–0.700).

**Conclusions:**

Our findings indicated that there is a cross-sectional association between participation in social activities and depressive symptoms among Chinese older adults, and the association differed between urban and rural older adults. This implies that participation in social activities may be significant for alleviating depressive symptoms of older adults. When encouraging older adults to participate in social activities, the government should consider urban-rural differences and take effective measures accordingly. Longitudinal studies are needed to examine the causal relationships between social activities and depressive symptoms among older adults.

**Supplementary Information:**

The online version contains supplementary material available at 10.1186/s12877-021-02541-y.

## Background

Depression is a mental health condition characterized by serious disruption of emotional equilibrium and a significant reduction in quality of life [1]. It has become a global public health problem and one of the most common illnesses among older adults [2]. The World Health Organization has reported that more than 264 million people worldwide experience depression [3], and the median global prevalence rate of depression among older adults has been reported to be approximately 10.3% [2]. Depression can cause several somatic and anxiety-related symptoms [4] and lead to cardiovascular risk [5]. Moreover, psychological and physical symptoms of depression can have a negative impact on quality of life among older adults [6]. The variety of presentations, unpredictable course and prognosis, and different responses to interventions lead to difficulties in depression treatment and symptom improvement [1]. Therefore, it is necessary to investigate the influencing factors of and interventions for depression among older adults. Existing studies have found that several factors, such as gender, marital status, educational background, income level, chronic disease(s), and relationships with their child/children may influence risk of depression among older adults [2, 7].

Participation in social activities has been found to help relieve depressive symptoms in older adults [8] and could be an indicator of successful aging [9]. Older adults and retirees living with an illness or disability are considered to engage in active aging when they continue to participate in society and contribute to their families, peers, and communities [10]. Participation in social activities helps older adults to adapt to changes in their lives, including physical activities [11] and leisure activities, such as playing cards and mahjong [12]. A few studies conducted globally have examined the correlation between social activity and health among older adults. For example, Croezen [13] found that social participation is associated with depressive symptoms among older adults in 10 European countries, and the strength of the association depends on the type of social activity. Roh [14] found that participation in physical, social, and religious activities is associated with lower odds for depression among Korean older adults. Further, the findings of a study in the United States of America investigated the relationship between participation in social activities and health among rural and urban older adults, which shows that creating an engaging environment has potential health benefits among the study population [15].

According to the National Bureau of Statistics of China, by the end of 2019, there were 254 million Chinese people aged 60 years and older, accounting for 18.1% of the country’s total population. With the rapid aging of Chinese population, older adults may face the challenge of age-related chronic illnesses, especially depression [16]. Thus, a large population of older adults could lead to an increased disease-related cost burden in China. Moreover, owing to cultural differences, the common types of social activities in China might differ from those in other countries [17]. Therefore, exploring the association between social activity and depressive symptoms among older adults could help promote active aging and reduce the prevalence of depression in China.

There are gaps in social and economic development between urban and rural regions in China [18], which may lead to a different prevalence of depression and differences in participation in social activities among older adults living in urban and rural regions. Studies have demonstrated that older adults in rural regions have higher levels of depressive symptoms than those in urban regions in China [18–20]. Liu et al. reported that 54.8% of Chinese older adults in urban regions participate in social activities, which is nearly 10% higher than their rural counterparts [21]. Therefore, the differences between urban and rural regions should be considered when exploring the association between social activities and depression among Chinese older adults. In addition, frequency is an important indicator in the measurement of social activity participation [17]. Previous studies found that either too frequent or too infrequent social activity participation is associated with negative impacts on older adults’ health [22, 23]. However, few studies have assessed the frequency of social activity participation and the impact of social activities on depressive symptoms among older adults. Thus, this study aimed to investigate the relationship between social activity participation and depressive symptoms among older adults in China, with a focus on urban-rural differences.

## Methods

### Data and sampling

The present study used data from the 2015 China Health and Retirement Longitudinal Study (CHARLS), conducted by the National School of Development of Peking University, using a multistage sampling method. A total of 21,096 residents from 12,400 households took part in the survey. Those aged 60 years and above who were able to communicate with the interviewers were eligible to participate (9785 individuals). Exclusive criteria included those with Alzheimer’s disease and severe psychiatric disorders other than depression (358 individuals) and those who did not provide available information on region, social activities and depressive symptoms (missing data of region: 32; missing data of social activities: 772; missing data of depressive symptoms: 1407. In total, 1530 subjects were excluded, and 8255 participants were included for analysis in our study.

Permission to use the data from the CHARLS was obtained by applying to the National School of Development of Peking University. All participants provided their informed consent for inclusion before taking part in the survey. As the original CHARLS protocol had been approved by the Ethical Review Committee of Peking University, ethical approval of the present data analysis was not needed. All methods in the present study were carried out in accordance with relevant guidelines and regulations.

### Assessment of depressive symptoms

Depressive symptoms were evaluated using the 10-item Center for Epidemiologic Studies Depression Scale (CES-D-10). The CES-D-10 has been validated for use in older Chinese populations and previously showed adequate validity and acceptable reliability [24]. Answers for the CES-D-10 include four choices related to frequency (days) per week: (1) rarely, (2) some (1–2), (3) occasionally (3–4), and (4) most of the time (5–7). Interviewees’ answers ranged from 0 (rarely) to 3 (most of the time) for negative questions, and 3 (rarely) to 0 (most of the time) for positive questions [25]. The scores ranged from 1 to 30 [26], a cutoff point of 10 was used to identify patients with depressive symptoms [27, 28].

### Assessment of social activities

The social activities were assessed by the CHARLS questionnaire, including interacting with friends, joining hobby groups (e.g., playing mahjong, chess, cards), taking part in sports groups (e.g., dancing, fitness, practicing Qigong), participating in community-related organizations, and taking part in volunteer activities [29]. Interviewees were asked whether they had engaged in any of the listed social activities in the past month. If they answered “yes,” they were further asked the frequency of their social activity participation in the past month. The answers were categorized as “not regularly,” “almost every week,” or “almost daily” [17].

### Other variables

Data were collected on demographic characteristics, including age, marital status, living status, educational background, and region. Marital status was divided into married/cohabitating and single. Single included divorced, separated, widowed, or never married. Living status was classified into living alone, just living with a spouse, and living with one’s child/children or others. Educational background was categorized as uneducated, primary school, middle school, and high school or above. The region was classified into urban and rural regions. Lifestyle factors included smoking status (current/former or never) and alcohol drinking (yes or no). Body mass index (BMI) was divided into normal body weight (18.5 ≤ BMI < 24 kg/m^2^), underweight (BMI < 18.5 kg/m^2^), overweight (24 ≤ BMI < 28 kg/m^2^), and obese (BMI ≥ 28 kg/m^2^), and the number of chronic diseases was categorized as none, one, or two or more. Satisfaction with the relationship with one’s child/children was categorized as satisfied or fair. Assessed activities of daily living (ADL) included eating, dressing, using the toilet, getting in and out of bed, defecating, and bathing. Participants who had any difficulty in any of the item were classified as having ADL function decline.

### Statistics analysis

Data were analyzed using the Statistical Package for the Social Sciences version 20.0 (IBM Corp., Armok, NY, USA). The respondents’ overall characteristics were described using means and standard deviations for continuous data and percentages for categorical data. A between-group comparison was performed using independent t-tests and χ2 test for continuous and categorical variables, respectively. The prevalence of depressive symptoms in different types and frequencies of social activity was analyzed by χ2 test. Multivariate logistic regression analysis was used to examine the association between social activity and depressive symptoms. Odds ratios (OR) and 95% confidence intervals (CI) were reported for the logistic regression model. Since there were not much differences between the results of the multivariate logistic regression analysis using the complete dataset and using the imputed dataset (all missing variables), it was assumed that missing data of social activities and depressive symptoms were missing completely at random, and we only selected those subjects who provided available information on social activities and depressive symptoms to conduct further analysis. Multiple imputations were performed using chained equations with 10 iterations to impute missing data [30, 31]. The percentage of missing data of each variable was shown in Tables 1 and 2. In the sensitivity analyses, multivariate logistic regression analysis was used to examine the association between social activities and depressive symptoms using multiple imputation technique (all missing variables) and using the dataset with complete cases (Stable 1 and Stable 2). Multiple linear regression analysis was used to examine the association between social activities and depressive symptoms (Stable 3). Statistical significance was set at *p* < 0.05.

**Table 1:** Characteristics of the study population

Characteristics	Total sample (*N* = 8255)	Urban (*N* = 1685)	Rural (*N* = 6570)	*χ*^*2*^*/t*	*P* value
Age, years, mean ± SD	67.85 ± 6.37	68.19 ± 6.65	67.76 ± 6.29	2.431	**0.012**
Missing, n(%)	0(0.0)	0(0.0)	0(0.0)		
Gender, n(%)				1.568	0.210
Male	4159(50.4)	826(49.0)	3333(50.7)		
Female	4096(49.6)	859(51.0)	3237(49.3)		
Missing, n(%)	0(0.0)	0(0.0)	0(0.0)		
Marital status, n(%)				0.180	0.672
Married/cohabitating	6386(77.4)	1310(77.7)	5076(77.3)		
Single	1869(22.6)	375(22.3)	1494(22.7)		
Missing, n(%)	0(0.0)	0(0.0)	0(0.0)		
Living status, n(%)				12.366	**0.002**
Living alone	996(12.1)	163(9.7)	833(20.1)		
Just living with a spouse	2077(25.2)	439(26.1)	1638(39.6)		
Living with child or others	2128(25.8)	458(27.2)	1670(40.3)		
Missing, n(%)	3054(37.0)	625(37.1)	2429(37.0)		
Educational background, n(%)				952.066	**< 0.001**
Uneducated	4202(50.9)	408(24.2)	3794(57.7)		
Primary school	1822(22.5)	337(20.0)	1485(22.6)		
Middle school	1116(13.5)	392(23.3)	724(11.0)		
High school and above	608(7.4)	338(20.1)	270(4.1)		
Missing, n(%)	507(6.1)	210(12.5)	297(4.5)		
Smoking status, n(%)				18.432	**< 0.001**
Current /Former	3657(44.3)	669(39.7)	2988(45.5)		
Never	4591(55.6)	1016(60.3)	3575(54.5)		
Missing, n(%)	7(0.1)	0(0.0)	7(0.1)		
Alcohol drinking, n(%)				1.249	0.264
Yes	2747(33.3)	580(34.4)	2167(33.0)		
No	5503(66.7)	1104(65.5)	4399(67.0)		
Missing, n(%)	5(0.1)	1(0.1)	4(0.1)		
BMI, n(%)				123.646	**< 0.001**
Normal	4196(50.8)	525(37.1)	3571(54.4)		
Underweight	506(6.1)	42(2.5)	464(7.1)		
Overweight	1836(22.2)	437(25.9)	1399(21.3)		
Obese	308(3.7)	86(5.1)	222(3.4)		
Missing, n(%)	1409(17.1)	495(29.4)	914(13.9)		
Number of chronic diseases, n(%)				12.954	**0.002**
0	1352(16.4)	217(12.9)	1135(17.3)		
1	2067(25.0)	393(23.3)	1674(25.5)		
≥ 2	3996(48.4)	819(48.6)	3177(48.4)		
Missing, n(%)	840(10.2)	256(15.2)	584(8.9)		
Satisfaction with relationship of child, n(%)				0.653	0.663
Satisfied	7197(87.2)	1461(86.7)	5736(87.3)		
Fair	301(3.6)	58(3.4)	241(3.7)		
Missing, n(%)	757(9.2)	166(9.9)	591(9.0)		
ADL, n(%)				50.545	**< 0.001**
Normal	4150(50.3)	898(53.3)	3252(49.5)		
Decline	2093(25.4)	296(17.6)	1797(27.4)		
Missing, n(%)	2012(24.4)	491(29.1)	1521(23.2)		
Depressive symptoms, n(%)				138.334	**< 0.001**
Yes	3099(37.5)	424(25.2)	2675(40.7)		
No	5156(62.5)	1261(74.8)	3895(59.3)		
Missing, n(%)	0(0.0)	0(0.0)	0(0.0)		

Note: The missing rate of variables were similar in the urban and rural sample except educational background, BMI, and number of chronic diseases. Percentages may not add up to 100% due to rounding

*SD* standard deviation, *BMI* body mass index, *ADL* activities of daily living

**Table 2:** Characteristics of type and frequency of social activities

Type and frequency of social activities	Total sample (*n* = 8255)	Urban (*n* = 1685)	Rural (*n* = 6570)	*χ*^*2*^	*P* value
Interaction with friends, (%)				25.369	**< 0.001**
No participation	5539(67.1)	1069(63.4)	4470(68.0)		
Not regularly	910(11.0)	222(13.2)	688(10.5)		
Almost every week	563(6.8)	148(8.8)	415(6.3)		
Almost daily	1243(15.1)	246(14.6)	997(15.2)		
Missing, n(%)	0(0.0)	0(0.0)	0(0.0)		
Hobby groups, (%)				99.373	**< 0.001**
No participation	6674(80.8)	1222(72.5)	5452(83.0)		
Not regularly	561(6.8)	148(8.8)	413(6.3)		
Almost every week	441(5.3)	138(8.2)	303(4.6)		
Almost daily	579(7.0)	177(10.5)	402(6.1)		
Missing, n(%)	0(0.0)	0(0.0)	0(0.0)		
Sports groups, (%)				325.055	**< 0.001**
No participation	7603(92.1)	1381(82.0)	6222(94.7)		
Not regularly	113(1.4)	33(2.0)	80(1.2)		
Almost every week	64(0.8)	33(2.0)	31(0.5)		
Almost daily	475(5.8)	238(14.1)	237(3.6)		
Missing, n(%)	0(0.0)	0(0.0)	0(0.0)		
Community-related organization, (%)				108.267	**< 0.001**
No participation	8073(97.4)	1582(93.9)	6455(98.2)		
Not regularly	135(1.6)	62(3.7)	73(1.1)		
Almost every week	53(0.6)	21(1.2)	32(0.5)		
Almost daily	30(0.4)	20(1.2)	10(0.2)		
Missing, n(%)	0(0.0)	0(0.0)	0(0.0)		
Volunteer, (%)				10.906	**0.012**
No participation	7075(85.5)	1410(83.7)	5647(86.0)		
Not regularly	895(10.8)	195(11.6)	700(10.7)		
Almost every week	168(2.0)	39(2.3)	129(2.0)		
Almost daily	135(1.6)	41(2.4)	94(1.4)		
Missing, n(%)	0(0.0)	0(0.0)	0(0.0)		

## Results

### Sample characteristics

Participants’ characteristics are shown in Table 1. In the present study, more than 50% of the older adults were male and most of the participants lived in rural regions. The mean age was 67.85 years (standard deviation = 6.37). Of the participants, 77.4% were married or cohabiting, 12.1% lived alone, and over half of the participants were uneducated. More than 50% of the participants had never smoked, 66.7% were not alcohol drinkers, and 50.8% had a normal BMI. Nearly 50% of the participants suffered from two or more chronic diseases, and 87.2% were satisfied with the relationship with their child/children. 50.3% of the participants had normal ADL function. The prevalence of depressive symptoms among older adults was 37.5% (25.2% in urban regions; 40.7% in rural regions, *p* <  0.001).

### Differences in social activity participation between urban and rural older adults

As Table 2 shows, 32.9% of the participants interacted with friends, 19.2% joined hobby groups, 7.9% took part in sports groups, 2.6% participated in community-related organizations, and 14.5% took part in volunteer activities. The proportion of urban older adults who participated in the five types of social activities mentioned above was higher than that of their rural counterparts (*p* <  0.05).

### Prevalence of depressive symptoms in different types and frequencies of social activities

As shown in Table 3, among urban older adults, there were significant differences in four types of social activities (interaction with friends, hobby groups, sport groups, and community-related organizations) and the prevalence of depressive symptoms. Among rural adults, there were significant differences in two social activities (hobby group and sport group) and the prevalence of depressive symptoms.

**Table 3:** Prevalence of depressive symptoms in different type and frequency of social activity participation

Type and frequency of social activities	Urban (%)		*χ*^*2*^	*P* value	Rural (%)		*χ*^*2*^	*P* value
	No	Yes			No	Yes		
Interaction with friends			13.603	**0.003**			4.837	0.184
No participation	61.3	69.8			67.0	69.5		
Not regularly	13.7	11.6			10.7	10.1		
Almost every week	10.0	5.2			6.5	6.1		
Almost daily	15.0	13.5			15.8	14.3		
Hobby groups			11.304	**0.010**			83.226	**< 0.001**
No participation	70.4	78.8			79.6	88.0		
Not regularly	9.4	7.1			7.2	4.9		
Almost every week	9.0	5.9			5.5	3.3		
Almost daily	11.3	8.3			7.7	3.8		
Sports groups			13.375	**0.004**			24.119	**< 0.001**
No participation	80.3	86.8			93.7	96.2		
Not regularly	1.7	2.6			1.3	1.2		
Almost every week	2.1	1.4			0.5	0.4		
Almost daily	15.8	9.2			4.5	2.3		
Community-related organization			10.293	**0.016**			6.478	0.091
No participation	92.9	96.9			97.9	98.7		
Not regularly	4.1	2.4			1.4	0.7		
Almost every week	1.5	0.5			0.5	0.4		
Almost daily	1.5	0.2			0.2	0.1		
Volunteer			3.563	0.316			2.080	0.556
No participation	83.0	85.8			85.4	86.7		
Not regularly	12.4	9.2			11.1	10.1		
Almost every week	2.4	2.1			2.0	1.9		
Almost daily	2.3	2.8			1.5	1.4		

### Multivariate logistic analysis

Table 4 shows the relationship between social activity type, frequency, and depressive symptoms. After adjusting for all covariates, among urban older adults, interacting with friends almost weekly or almost daily were both negatively associated with depressive symptoms (almost weekly: OR = 0.568, 95% CI: 0.337–0.955, *p* = 0.033; almost daily: OR = 0.664, 95%CI: 0.453–0.973, *p* = 0.036), as were taking part in sports almost daily (OR = 0.664, 95%CI: 0.445–0.991, *p* = 0.045) and participating in a community-related organization almost daily (OR = 0.107, 95%CI: 0.012–0.952, *p* = 0.045). Among rural older adults, interacting with friends almost daily was negatively associated with depressive symptoms (OR = 0.847, 95%CI: 0.720–0.996, *p* = 0.044), as was participating in hobby groups almost weekly and almost daily (almost weekly: OR = 0.683, 95%CI: 0.518–0.902, *p* = 0.007; almost daily: OR = 0.567, 95%CI: 0.440–0.731, *p <* 0.001) and taking part in sports almost daily (OR = 0.506, 95%CI: 0.366–0.700, *p <* 0.001). In the sensitivity analyses, we did not observe much difference between the results of the multiple linear regression model (STable1).

**Table 4:** Multiple logistic regression model testing the association between social activities and depressive symptoms among urban and rural older adults

Social activities	Urban			Rural		
	OR	95% CI	*P* value	OR	95% CI	*P* value
Interacting with friends (ref. no participant)						
Not regularly	0.888	0.600–1.315	0.554	0.929	0.771–1.118	0.436
Almost every week	0.568	0.337–0.955	**0.033**	0.995	0.788–1.256	0.966
Almost daily	0.664	0.453–0.973	**0.036**	0.847	0.720–0.996	**0.044**
Hobby groups (ref. no participant)						
Not regularly	0.728	0.459–1.155	0.178	0.811	0.641–1.025	0.080
Almost every week	0.838	0.503–1.397	0.498	0.683	0.518–0.902	**0.007**
Almost daily	0.847	0.549–1.308	0.454	0.567	0.440–0.731	**< 0.001**
Sports groups (ref. no participant)						
Not regularly	1.578	0.680–3.661	0.288	0.893	0.540–1.477	0.660
Almost every week	0.707	0.263–1.903	0.493	0.783	0.343–1.785	0.560
Almost daily	0.664	0.445–0.991	**0.045**	0.506	0.366–0.700	**< 0.001**
Community-related organization (ref. no participant)						
Not regularly	0.863	0.404–1.844	0.704	0.876	0.501–1.533	0.644
Almost every week	0.593	0.126–2.787	0.508	0.894	0.390–2.053	0.792
Almost daily	0.107	0.012–0.952	**0.045**	0.827	0.205–3.337	0.790
Volunteer (ref. no participant)						
Not regularly	0.977	0.638–1.497	0.917	1.072	0.894–1.286	0.453
Almost every week	2.229	0.912–5.450	0.079	1.144	0.771–1.700	0.504
Almost daily	2.165	0.966–4.854	0.061	1.066	0.667–1.703	0.789
Age	0.987	0.969–1.006	0.188	0.984	0.974–0.993	**0.001**
Gender (ref. male)						
female	1.435	0.995–2.069	0.053	2.046	1.735–2.412	**< 0.001**
Marital status (ref. Married/cohabitating)						
Single	1.553	1.036–2.328	**0.033**	1.277	1.043–1.564	**0.019**
Living status (ref. Living alone)						
Just living with spouse	0.900	0.495–1.636	0.725	0.757	0.578–0.993	**0.044**
Living with child or others	1.346	0.786–2.304	0.272	0.835	0.686–1.018	0.075
Educational level (ref. Uneducated)						
Primary school	0.794	0.557–1.132	0.201	0.819	0.715–0.937	0.004
Middle school	0.694	0.487–0.991	**0.045**	0.596	0.489–0.727	**< 0.001**
High school and above	0.540	0.369–0.788	**0.001**	0.552	0.396–0.769	**< 0.001**
Smoking status (ref. Current /Former)						
Never	0.814	0.577–1.147	0.240	0.860	0.736–1.005	0.058
Alcohol drinking (ref. Yes)						
No	1.129	0.837–1.525	0.427	1.046	0.920–1.190	0.490
BMI (ref. Normal)						
Underweight	0.589	0.253–1.372	0.204	1.509	1.204–1.890	**< 0.001**
Overweight	0.589	0.408–0.850	**0.006**	0.769	0.665–0.889	**< 0.001**
Obese	0.812	0.487–1.356	0.423	0.604	0.426–0.857	**0.006**
ADL (ref. Completely normal)						
Functional decline	3.314	2.355–4.662	**< 0.001**	2.906	2.575–3.279	**< 0.001**
Chronic diseases (ref. 0)						
1	1.765	1.090–2.859	**0.021**	1.566	1.277–1.920	**< 0.001**
≥ 2	3.289	2.104–5.141	**< 0.001**	2.391	2.015–2.837	**< 0.001**
Satisfaction with relationship of child (ref. Satisfied)						
Fair	1.930	1.034–3.603	**0.039**	1.151	0.861–1.537	0.340

Note: *OR* Odd ratio, *CI* Confidence Interval, *BMI* body mass index, *ADL* activities of daily living

## Discussion

The present study investigated the association between social activities and depressive symptoms among urban and rural older adults in China. The findings showed that, compared with their urban counterparts, rural older adults had a higher prevalence of depressive symptoms, which is consistent with previous studies [18–20]. This finding is likely to be related to the differences in economic status and health levels among rural and urban older adults in China. The gap in economic development may lead to differences in social welfare between rural and urban regions [19]. Urban older adults usually live under good economic conditions and have access to basic financial resources, such as retirement pensions and a minimum living allowance, which could meet their living requirements and provide them with a sense of security [20]. In contrast, rural older adults generally have no regular income and mainly receive financial resources from agriculture. Li [32] reported that rural older adults might face stress related to relatively lower income in the rural regions of China. Low income is one of the main causes of depression among older adults [2]. Furthermore, physical health is an important basis for mental health [1]. Older adults often experience declines in physical function which severely influence their normal lives and lead to negative emotions, such as loneliness and disappointment [33]. However, rural older adults need to engage in agricultural work to increase their income, and long-term hard work might damage their health [21]. Both economic distress and poor health status may lead to higher anxiety levels in rural older adults, which could cause tendencies toward negative emotions and a higher prevalence of depressive symptoms.

Our study found that the proportion of social activity participation in rural older adults was significantly lower than that of their urban counterparts, which is consistent with other studies [20, 21]. This might be explained by the imbalance in socioeconomic development between urban and rural regions. The infrastructure of older adult services plays an important role in social activity participation, which is relatively undeveloped in rural regions. Lin [21] reported that the frequency of participation in social activities among older adults is determined by the convenience of access to activity centers. A previous study in China showed that 3.36% of urban older adults rated their community infrastructure as the most deficient, while the corresponding rate was 31.4% among rural older adults [20]. A livable community environment can provide safe, convenient, and comfortable support for older adults to participate in their community; however, human and material resources in rural communities are limited and cannot provide sufficient support for older adults to engage in social activities [18]. Overall, the gap in infrastructure and community services between urban and rural regions in China may lead to unequal opportunities for older adults to participate in social activities [19].

Our results showed that interacting with friends and taking part in sports groups were negatively associated with depressive symptoms in both urban and rural older adults, which indicated that these activities may relieve depressive symptoms in this population. Interacting with friends can protect against depression by providing stress-coping resources [34]. With their increased age and physical function decline, older adults inevitably reduce or lose their social relationships. Actively interacting with friends can help them increase their personal relationships and receive support to ease their negative emotions. The benefits of exercise in reducing depressive symptoms have been demonstrated, as physical activity protects mental health through biological and psychosocial mechanisms, suggesting that exercise is an important form of social participation for older adults in China [11, 16]. Exercise itself has health-promoting effects, offering many physical, mental, and cognitive benefits for older adults [16], which can improve their physical condition, promote physical function recovery, and alleviate physical discomfort. A previous study demonstrated that engaging in exercises that meet physical activity guidelines can provide potential benefits by reducing depression risk, leading to a 40% reduction in the likelihood of reporting concurrent depression [35]. Furthermore, participating in sports groups can provide social communication opportunities among older adults. By interacting with others, they could relax and receive emotional support, which could help them to reduce their depressive symptoms [29].

Our results also showed that differences in the frequency of interacting with friends had a different association with depressive symptoms between urban and rural older adults. Interacting with friends almost weekly was negatively associated with depressive symptoms in urban older adults, while for rural older adults, only almost daily interaction with friends was negatively associated with depressive symptoms. This might be explained by differences in interpersonal networks due to lifestyle variations in urban and rural regions in China. Close interpersonal networks in rural regions lead to people communicating with others easily and frequently; whereas in urban regions, interpersonal relationships are more open with less frequent contact [36]. The reality in China is that rural older adults typically reside in the same village as almost all their friends, which is convenient for social interaction, while urban older adults may have to spend more time and energy to meet their friends frequently. Furthermore, we found that only taking part in sports almost daily was negatively associated with depressive symptoms among both urban and rural older adults, which indicated a possible dose-effect relationship between social activity and depressive symptoms. Thus, the frequency of social activity participation should be considered when taking measures to ameliorate depressive symptoms in older adults.

Our findings also showed that there were no significant correlations between participating in community-related organizations and depressive symptoms among rural older adults. A possible reason for this could be the differences in community infrastructure between urban and rural regions in China. Community participation plays an important role in the impact of social activities on health, and active participation in community activities is beneficial for increasing the sense of community among older adults, thereby affecting their health status [9]. One study found that communities with good infrastructure could promote social interactions among older adults, resulting in their experiencing fewer depressive symptoms [20]. As physical environment quality affects mental health [37], urban communities with better infrastructure can actively organize older adults to participate in social activities which might relieve their depressive symptoms. In rural communities with insufficient infrastructure, public sports facilities being suitable for use by older adults are needed.

In addition, hobbies, such as playing mahjong or cards, along with other leisure activities, can provide opportunities for social contact among older adults, which have been shown to prevent loneliness [12]. However, our study found that hobby group participation was negatively associated with depressive symptoms among rural, but not urban older adults. A possible explanation for this phenomenon is that, as mentioned above, urban communities have enough human and financial resources to provide space and facilities for older adults to participate in hobby groups, thereby enriching the lives of urban older adults and alleviating their loneliness [38]. Therefore, participating in hobby groups could become a part of daily life among urban older adults, which could weaken the influence of these groups on depressive symptoms. Another explanation could be that, because public facilities and community services are more developed in urban regions compared with rural regions, urban older adults have more options to reduce their depressive symptoms. For example, urban medical institutions have been successful in providing health education for urban older adults and addressing mental health problems in various ways, such as providing psychological consultation services. In rural regions, when people were young and strong, they focused on making money to support their families and spent little time enjoying themselves, especially through participating in recreational activities. Joining a hobby group may help them experience new and exciting activities, which could have a positive impact on their depressive symptoms.

The present study has some limitations. First, because it was a cross-sectional study, the causal relationships between social activities and depressive symptoms among older adults in both rural and urban regions could not be investigated. Participants’ depressive symptoms might have been a hindrance to their participation in social activities. Second, the survey depended on self-reports, which could lead to the risk of recall bias owing to false or inaccurate responses from some participants. Third, the CHARLS could not assess the quality of interpersonal interactions during social activities, which should be considered in future studies. Fourth, the prevalence of depressive symptoms was >10% (25.2% in urban regions; 40.7% in rural regions) in our study. Therefore, the OR derived from the logistic regression can no longer approximate the risk ratio [39]. Fifth, we did not divide volunteer activity into formal volunteering and informal volunteering in this study, the relationships between formal and informal volunteer activity and depressive symptoms among older adults need further study. Finally, due to the low frequency of participation in community-related organizations, the relationship between participation in community-related organizations and depressive symptoms among older adults may not be an adequate estimate.

## Conclusion

In the present study, rural older adults had a higher prevalence of depressive symptoms and participated in fewer social activities, compared with their counterparts in urban regions. Both interacting with friends and participating in sports groups were negatively associated with depressive symptoms in both urban and rural older adults. However, participating in community-related organizations was only found to reduce depressive symptoms among urban older adults, while joining hobby groups decreased depressive symptoms only among rural older adults. There are different associations between different frequencies of social activity participation and the prevalence of depressive symptoms among urban and rural older adults. Longitudinal studies are needed to examine the causal relationships between social activities and depressive symptoms.

### Policy implications

Although we can not investigate the causal relationships between social activities and depressive symptoms among older adults in both rural and urban regions, this study could provide some recommendations. We recommend that the government take effective measures to encourage older adults to participate in social activities. In urban regions, the benefits of social activity participation, such as interacting with friends and participating in community organizations, should be highlighted through community health education. The frequency and type of social activity participation should be taken into consideration. In rural regions, because of the high prevalence of depressive symptoms and low level of social activity participation, essential financial support, basic facilities, and platforms for rural older adults should be provided to help them participate in social activities easily. Communities should strengthen their organizational capabilities, establish standardized community organizations, and expand the channels for older adults to join in social activities.

## Supplementary Information

**Additional file 1: STable 1.** Associations between social activities and depressive symptoms among urban and rural older adults using a dataset with multiple imputation techniques (all missing variables). **STable 2.** Associations between social activities and depressive symptoms among urban and rural older adults using a dataset with complete cases. **STable 3.** Multiple linear regression model testing the association between social activities and depressive symptoms.

## Data Availability

The datasets analysed for the current study are available in the CHARLS (http://charls.pku.edu.cn/zh-CN/page/data).

## Abbreviations

ADL: Activities of daily living
BMI: Body mass index
CES-D 10: 10-item Center for Epidemiologic Studies Depression Scale
CHARLS: China Health and Retirement Longitudinal Study
CI: Confidence intervals
OR: Odds ratios
