# Supplementary material for: Urban-rural differences in the association between social activities and depressive symptoms among older adults in China: a cross-sectional study
Source: BMC Geriatr. 2021 Oct 18;21:569. doi: 10.1186/s12877-021-02541-y (PMC8522037; doi:10.1186/s12877-021-02541-y)
Supplement: Supplementary file 1 — Additional file 1: STable 1. Associations between social activities and depressive symptoms among urban and rural older adults using a dataset with multiple imputation techniques (all missing variables). STable 2. Associations between social activities and depressive symptoms among urban and rural older adults using a dataset with complete cases. STable 3. Multiple linear regression model testing the association between social activities and depressive symptoms. [file 12877_2021_2541_MOESM1_ESM.docx]

STable 1 Associations between social activities and depressive symptoms among urban and rural older adults using a dataset with multiple imputation techniques (all missing variables)

| Social activities | Urban | | | Rural | | |
| --- | --- | --- | --- | --- | --- | --- |
|  | OR | 95% CI | *P* value | OR | 95% CI | *P* value |
| Interacting with friends (ref. no participant) |  |  |  |  |  |  |
| Not regularly | 0.930 | 0.654-1.323 | 0.687 | 0.897 | 0.754-1.068 | 0.223 |
| Almost every week | 0.621 | 0.385-1.004 | 0.052 | 0.940 | 0.758-1.166 | 0.574 |
| Almost daily | 0.563 | 0.399-0.739 | **0.001** | 0.823 | 0.711-0.951 | **0.008** |
| Hobby groups (ref. no participant) |  |  |  |  |  |  |
| Not regularly | 0.660 | 0.425-1.027 | 0.065 | 0.977 | 0.783-1.218 | 0.835 |
| Almost every week | 0.868 | 0.551-1.368 | 0.543 | 0.684 | 0.527-0.887 | **0.004** |
| Almost daily | 0.851 | 0.573-1.265 | 0.426 | 0.592 | 0.467-0.749 | **< 0.001** |
| Sports groups (ref. no participant) |  |  |  |  |  |  |
| Not regularly | 1.960 | 0.989-3.883 | 0.054 | 0.826 | 0.554-1.234 | 0.351 |
| Almost every week | 1.097 | 0.441-2.731 | 0.842 | 1.031 | 0.479-2.219 | 0.938 |
| Almost daily | 0.914 | 0.643-1.300 | 0.618 | 0.482 | 0.355-0.654 | **< 0.001** |
| Community-related organization (ref. no participant) |  |  |  |  |  |  |
| Not regularly | 0.643 | 0.356-1.159 | 0.142 | 0.811 | 0.469-1.404 | 0.455 |
| Almost every week | 0.334 | 0.075-1.477 | 0.148 | 0.838 | 0.362-1.937 | 0.679 |
| Almost daily | 0.094 | 0.012-0.749 | **0.026** | 0.799 | 0.610-1.046 | 0.103 |
| Volunteer (ref. no participant) |  |  |  |  |  |  |
| Not regularly | 0.906 | 0.595-1.379 | 0.645 | 1.000 | 0.842-1.188 | 0.999 |
| Almost every week | 1.827 | 0.955-3.497 | 0.069 | 0.910 | 0.666-1.243 | 0.554 |
| Almost daily | 1.761 | 0.941-3.296 | 0.077 | 1.338 | 0.893-2.004 | 0.158 |

Note: Model : adjusted for age, gender, marital status, living status, education background, smoking status, alcohol drinking, number of chronic diseases, body mass index, satisfaction with the relationship with the child, and activities of daily living.

STable 2 Associations between social activities and depressive symptoms among urban and rural older adults using a dataset with complete cases

| Social activities | Urban | | | Rural | | |
| --- | --- | --- | --- | --- | --- | --- |
|  | OR | 95% CI | *P* value | OR | 95% CI | *P* value |
| Interacting with friends (ref. no participant) |  |  |  |  |  |  |
| Not regularly | 0.807 | 0.540-1.206 | 0.296 | 0.883 | 0.735-1.060 | 0.182 |
| Almost every week | 0.547 | 0.323-0.928 | **0.025** | 0.897 | 0.709-1.135 | 0.365 |
| Almost daily | 0.762 | 0.525-1.107 | 0.154 | 0.799 | 0.681-0.937 | **0.006** |
| Hobby groups (ref. no participant) |  |  |  |  |  |  |
| Not regularly | 0.653 | 0.406-1.049 | 0.078 | 0.694 | 0.547-0.881 | **0.003** |
| Almost every week | 0.740 | 0.452-1.209 | 0.229 | 0.631 | 0.475-0.838 | **0.002** |
| Almost daily | 0.808 | 0.519-1.259 | 0.347 | 0.503 | 0.390-0.650 | **< 0.001** |
| Sports groups (ref. no participant) |  |  |  |  |  |  |
| Not regularly | 1.529 | 0.695-3.360 | 0.291 | 0.815 | 0.490-1.355 | 0.431 |
| Almost every week | 0.588 | 0.213-1.629 | 0.307 | 0.707 | 0.304-1.646 | 0.421 |
| Almost daily | 0.620 | 0.417-0.922 | **0.018** | 0.469 | 0.340-0.646 | **< 0.001** |
| Community-related organization (ref. no participant) |  |  |  |  |  |  |
| Not regularly | 0.661 | 0.305-1.430 | 0.293 | 0.813 | 0.449-1.470 | 0.493 |
| Almost every week | 0.562 | 0.122-2.519 | 0.460 | 0.933 | 0.405-2.150 | 0.870 |
| Almost daily | 0.136 | 0.017-1.074 | 0.058 | 0.611 | 0.155-2.405 | 0.481 |
| Volunteer (ref. no participant) |  |  |  |  |  |  |
| Not regularly | 0.989 | 0.637-1.537 | 0.961 | 1.004 | 0.835-1.206 | 0.969 |
| Almost every week | 2.183 | 0.944-5.046 | 0.068 | 1.206 | 0.811-1.793 | 0.355 |
| Almost daily | 1.841 | 0.833-4.070 | 0.132 | 1.259 | 0.791-2.006 | 0.332 |

Note: Model : adjusted for age, gender, marital status, living status, education background, smoking status, alcohol drinking, number of chronic diseases, body mass index, satisfaction with the relationship with the child, and activities of daily living.

STable 3 Multiple linear regression model testing the association between social activities and depressive symptoms

|  | Social activities | Unstandardized Coefficients | | *t* | *P* value | 95% confidence interval | |
| --- | --- | --- | --- | --- | --- | --- | --- |
|  |  | B | SE |  |  | Lower bound | Upper bound |
| Urban | Interacting with friends (ref. no participant) |  |  |  |  |  |  |
|  | Not regularly | -0.295 | 0.424 | -0.695 | 0.487 | -1.125 | 0.536 |
|  | Almost every week | -1.512 | 0.494 | -3.062 | **0.002** | -2.480 | -0.544 |
|  | Almost daily | -0.742 | 0.419 | -1.771 | 0.077 | -1.567 | 0.082 |
|  | Hobby groups (ref. no participant) |  |  |  |  |  |  |
|  | Not regularly | -0.385 | 0.480 | -0.802 | 0.422 | -1.326 | 0.556 |
|  | Almost every week | 0.203 | 0.498 | 0.407 | 0.684 | -0.733 | 1.179 |
|  | Almost daily | -0.613 | 0.443 | -1.385 | 0.166 | -1.481 | 0.255 |
|  | Sports groups (ref. no participant) |  |  |  |  |  |  |
|  | Not regularly | 1.040 | 0.985 | 1.056 | 0.291 | -0.891 | 2.971 |
|  | Almost every week | -0.783 | -0.976 | -0.802 | 0.423 | -2.697 | 1.131 |
|  | Almost daily | -0.847 | 0.399 | -2.121 | **0.034** | -1.631 | -0.063 |
|  | Community-related organization (ref. no participant) |  |  |  |  |  |  |
|  | Not regularly | -0.416 | 0.739 | -0.563 | 0.574 | -1.865 | 1.034 |
|  | Almost every week | -1.207 | 1.226 | -0.985 | 0.325 | -3.610 | 1.197 |
|  | Almost daily | -3.329 | 1.261 | -2.640 | **0.008** | -5.802 | -0.855 |
|  | Volunteer (ref. no participant) |  |  |  |  |  |  |
|  | Not regularly | 0.029 | 0.436 | 0.068 | 0.946 | -0.825 | 0.884 |
|  | Almost every week | 0.951 | 0.911 | 1.044 | 0.297 | -0.836 | 2.738 |
|  | Almost daily | 0.348 | 0.880 | 0.396 | 0.692 | -1.377 | 2.073 |
| Rural | Interacting with friends (ref. no participant) |  |  |  |  |  |  |
|  | Not regularly | -0.088 | 0.263 | -0.335 | 0.738 | -0.605 | 0.428 |
|  | Almost every week | - 0.415 | 0.321 | -1.294 | 0.196 | -1.044 | 0.214 |
|  | Almost daily | -0.556 | 0.224 | -2.489 | **0.013** | -0.995 | -0.118 |
|  | Hobby groups (ref. no participant) |  |  |  |  |  |  |
|  | Not regularly | -0.748 | 0.319 | -2.349 | **0.019** | -1.373 | -0.124 |
|  | Almost every week | -1.029 | 0.369 | -2.790 | **0.005** | -1.752 | -0.306 |
|  | Almost daily | -1.602 | 0.322 | -4.978 | **< 0.001** | -2.233 | -0.971 |
|  | Sports groups (ref. no participant) |  |  |  |  |  |  |
|  | Not regularly | -0.229 | 0.695 | -0.329 | 0.742 | -1.590 | 1.133 |
|  | Almost every week | -1.443 | 1.114 | -1.296 | 0.195 | -3.627 | 0.740 |
|  | Almost daily | -1.685 | 0.415 | -4.056 | **< 0.001** | -2.500 | -0.870 |
|  | Community-related organization (ref. no participant) |  |  |  |  |  |  |
|  | Not regularly | 0.124 | 0.743 | 0.167 | 0.867 | -1.333 | 1.582 |
|  | Almost every week | -1.136 | 1.101 | -1.032 | 0.302 | -3.294 | 1.022 |
|  | Almost daily | -1.565 | 1.959 | -0.799 | 0.424 | -5.405 | 2.275 |
|  | Volunteer (ref. no participant) |  |  |  |  |  |  |
|  | Not regularly | 0.062 | 0.251 | 0.246 | 0.806 | -0.431 | 0.555 |
|  | Almost every week | 0.080 | 0.552 | 0.146 | 0.884 | -1.001 | 1.162 |
|  | Almost daily | -0.078 | 0.644 | -0.120 | 0.904 | -1.339 | 1.184 |

Note: B, Coefficient; SE, Standard error. Model : adjusted for age, gender, marital status, living status, education background, smoking status, alcohol drinking, number of chronic diseases, body mass index, satisfaction with the relationship with the child, and activities of daily living.
